# Development and validation of direct assay for cholesterol content of erythrocytes

**DOI:** 10.1042/BSR20253335

**Published:** 2025-07-17

**Authors:** Azusa Yamazaki, Yuna Hakii, Akira Yoshimoto, Takahiro Kameda, Naoya Ichimura, Shuji Tohda, Ryunosuke Ohkawa

**Affiliations:** 1 Clinical Bioanalysis and Molecular Biology, Graduate School of Medical and Dental Sciences, Institute of Science Tokyo, 1-5-45 Yushima, Bunkyo-ku, Tokyo, 113-8510, Japan; 2 Clinical Laboratory, Institute of Science Tokyo hospital, 1-5-45 Yushima, Bunkyo-ku, Tokyo, 113-8510, Japan; 3 Clinical Laboratory Science, Faculty of Medical Technology, Teikyo University, 2-11-1 Kaga, Itabashi-ku, Tokyo, 173-8605, Japan

**Keywords:** atherosclerotic cardiovascular disease, biomarker, enzymatic assay, erythrocyte cholesterol, lipid metabolism

## Abstract

Erythrocytes contain a significant amount of membrane cholesterol, which is continuously exchanged with lipoproteins. Recent studies suggest that erythrocyte cholesterol content correlates positively with atherosclerotic cardiovascular disease (ASCVD) severity independent of low-density lipoprotein levels, potentially reflecting residual ASCVD risk. However, conventional methods for measuring erythrocyte cholesterol content require labor-intensive lipid extraction procedures, limiting their clinical applicability. In this study, we developed a novel enzymatic assay that enables direct quantification of erythrocyte total cholesterol content using two denaturants to eliminate hemoglobin interference. This simple method demonstrated high accuracy and precision, as confirmed by intra-assay repeatability, between-day precision, linearity, and spike-recovery tests. Using this assay, we determined the erythrocyte cholesterol content per volume (154.8 ± 2.9 mg/dl in men, 155.9 ± 6.9 mg/dl in women) and per cell (139.0 ± 5.2 fg/cell in men, 140.8 ± 5.3 fg/cell in women) (*n* = 12, healthy subjects). While erythrocyte cholesterol content per volume correlated with the conventional method, the erythrocyte cholesterol content per cell showed no such correlation. Moreover, neither measure was associated with serum lipid levels, suggesting their potential as independent biomarkers for ASCVD. Additionally, we evaluated erythrocyte cholesterol content across different maturation stages and found that older erythrocytes had significantly lower cholesterol content, consistent with mass spectrometry results. These findings further validated the physiological relevance of the proposed method. In conclusion, we successfully established a simple and clinically applicable enzymatic method for measuring erythrocyte cholesterol content, providing novel insights into erythrocyte cholesterol metabolism and its potential role in ASCVD risk assessment.

## Introduction

Serum cholesterol concentrations in low-density lipoprotein cholesterol (LDL-C) and high-density lipoprotein cholesterol (HDL-C) have been widely measured to assess the risk of atherosclerotic cardiovascular disease (ASCVD). While LDL-C lowering therapy significantly reduces the incidence of ASCVD events, a substantial proportion of patients continue to experience adverse outcomes, indicating residual risk [1]. Although this approach is beneficial, it is important to acknowledge that it does not completely eliminate ASCVD risk. Consistent with the various anti-atherosclerotic effects of HDL, numerous studies dating back several decades have demonstrated that patients with low levels of HDL-C are at an increased risk of ASCVD events [2,3]. However, HDL-C level may not be a reliable predictor of residual risk in patients already receiving statin therapy [4]. In addition, higher HDL-C levels have been rather associated with higher cardiovascular mortality [5]. This highlights the need for novel biomarkers that can provide a more comprehensive assessment of individual ASCVD risk and identify patients who may benefit from more intensive risk management strategies, potentially enabling personalized treatment approaches.

Erythrocytes, which make up approximately half of the blood volume, have the highest molar cholesterol ratio among all lipids they contain and exhibit cholesterol concentrations closely resembling those of serum [6,7]. Previously, we reported that the incubation of erythrocytes with plasma facilitated significant transfer of cholesterol from erythrocytes to HDL, while promoting cholesterol transfer from LDL to erythrocytes [8]. Furthermore, erythrocytes contribute to reverse cholesterol transport by receiving cholesterol from foam cells through both HDL and free apolipoprotein A-I [9]. Collectively, these results indicate that erythrocytes play a crucial role in cholesterol homeostasis and may significantly contribute to the development of atherosclerosis.

In addition, because most of the cholesterol in erythrocytes is distributed on the membrane, the cholesterol content of erythrocyte membranes (CEM) has been measured as an index of erythrocyte-related cholesterol. A previous study reported that CEM was higher in patients with coronary artery stenosis than in those with chronic stable angina, suggesting that CEM is associated with vulnerable plaques [10–12]. Notably, several studies suggest that CEM is positively correlated with the severity of ASCVD, a correlation that persists regardless of LDL-C levels [13–15].

Although the measurement of CEM could potentially serve as a surrogate marker for predicting residual risk, its clinical application is limited primarily because conventional CEM measurement methods are laborious and time-consuming. Furthermore, significant inconsistencies exist across studies regarding methodology, including variations in erythrocyte membrane separation techniques, lipid extraction protocols, and the accuracy of the measured values.

This study aimed to develop a simplified and clinically applicable method for measuring erythrocyte cholesterol content, evaluate its accuracy, and assess the feasibility of an improved method for clinical application. Using this method, we measured cholesterol content in healthy subjects and analyzed the cholesterol content of erythrocyte fractions at different maturation stages in each participant.

## Materials and methods

### Blood samples

Blood samples were obtained from 17 healthy volunteers, who provided written informed consent to participate in the study. This study was approved by the Ethics Committee of the Faculty of Medicine, Tokyo Medical and Dental University (currently the Institute of Science Tokyo) (Approval No. M2000-1790).

### Sample preparation

Whole blood samples were collected into EDTA-2K-coated tubes and centrifuged at 350×g for 15 min at 4°C without deceleration. After centrifugation, the upper layer containing plasma and buffy coat was carefully removed. The remaining erythrocytes were washed four times with saline by centrifugation at 2,000×g for 5 min at 4°C to obtain purified packed erythrocytes. The effectiveness of this washing method in efficiently removing plasma-derived cholesterol from the packed erythrocytes was confirmed by two approaches. First, we observed no significant difference in the cholesterol content of the washing supernatant between the third and fourth washes of erythrocytes from 12 healthy subjects (Supplementary Figure S1A). Second, to account for variations in hematocrit, we prepared three types of whole blood with differing hematocrit levels (60%, 40%, and 20%) from four different individuals. After similarly washing these samples, we confirmed that there was no significant difference in the cholesterol content of the washing supernatant among the three hematocrit levels (Supplementary Figure S1B). To prepare the hemoglobin solution, packed erythrocytes were hemolyzed by hypotonic treatment with four times the volume of 5 mM Na_2_HPO_4_ (pH 8.0). The hemolysate was then centrifuged at 20,630×g for 30 min at 4°C to remove cellular debris. To obtain serum samples, whole blood in procoagulant tubes was centrifuged at 2,000×g for 15 min at 4°C after allowing the blood to clot for 15 min. All samples, including packed erythrocytes, hemoglobin solutions, and serum samples, were stored at −80°C until use.

### Measurement of erythrocytes total cholesterol content

Erythrocyte cholesterol content was determined using a novel direct enzymatic method designed to eliminate hemoglobin interference. This was achieved by adding sodium dodecyl sulfate (SDS) and sodium nitrite (NaNO_2_) to the packed erythrocytes at final concentrations of 1% and 10 mM, respectively. To validate the feasibility of this approach, we first confirmed that these concentrations of SDS and NaNO_2_ did not interfere with the subsequent cholesterol measurement system (Supplementary Figure S2). In addition to these additives, packed erythrocytes were diluted 10-fold with 5 mM Na_2_HPO_4_ (pH 8.0). These samples were mixed with the first reagent for cholesterol measurement “T-CHO (S)” (Denka, Tokyo, Japan) at a 1:9 ratio and incubated at 37°C for 20 min. After incubation, the absorbances at 555 and 600 nm were measured using a Sunrise Rainbow microplate absorbance reader (Tecan Japan Co., Tokyo, Japan). The mixture and the second reagent were then combined at a 10:3 ratio. Following a second incubation under the same conditions, absorbance was measured at 600 nm. Cholesterol concentration was determined using a standard curve based on the difference in absorbance at 600 nm between the first and second measurements. Since this reagent contains cholesterol esterase in the first reagent, the measured value is total cholesterol (TC), which is the sum of esterified and free cholesterol. The cholesterol standard solution was prepared using ‘Cholesterol standard’ (C_27_H_45_OH, MW = 386.65) (FUJIFILM Wako Pure Chemical Corporation, Osaka, Japan). Cholesterol standard (0.3 g) was added to 5 ml TritonX-100 and dissolved by stirring while heating. Subsequently, 90 ml of distilled water was gently added, and after further stirring, the mixture was boiled in a heater for 60 s. After cooling in running water with gentle stirring, 4 g of sodium cholate was added and completely dissolved. Finally, the solution was brought to a final volume of 100 ml with distilled water, filtered through a 0.22 µm filter, frozen in small aliquots, stored at −80°C, and thawed as needed for each measurement. The cholesterol concentration in the stock standard solution was verified using an enzymatic method in the laboratory. A calibration curve was generated using serial dilutions of the standard solutions.

### Measurement of the absorption difference spectrum

Samples were mixed with the first reagent and incubated under the conditions described above. The absorption spectrum of the mixture was measured from 450 to 750 nm using a UV-visible spectrophotometer (UV-1280, Shimadzu Co., Kyoto, Japan). Subsequently, a second reagent was added to the mixture, and after incubation under the same conditions, the absorption spectrum was measured again at the same wavelength range. The absorption difference spectrum was calculated by subtracting the first spectrum from the second spectrum after an appropriate volume correction. This difference represents the absorption spectrum of the quinone pigment generated by the enzymatic reaction.

### Validation of the new direct method

We conducted a validation study for the newly established direct method. Intra-assay repeatability was evaluated by measuring the TC concentrations in three distinct packed erythrocyte samples, with each sample measured in 20 replicates from the sample dilution step. Between-day precision was assessed by measuring three other different samples over 20 consecutive days. For each measurement day, a new set of three aliquots, frozen as packed erythrocytes at −80°C, was thawed and analyzed. Calibration samples were measured daily to generate calibration curves. To evaluate the linearity, cholesterol standard solutions were serially diluted and spiked into hemoglobin solutions to prepare a series of samples with varying cholesterol concentrations. The hemoglobin concentration in these spiked samples was adjusted to 3,500 mg/dl. TC concentration was measured in triplicate for each sample. Accuracy was assessed by performing a spike-recovery test. Known amounts of cholesterol standard solution were spiked into packed erythrocytes within the linear range of the assay. The TC concentration in the spiked samples was measured in triplicate, and the recovery rates were calculated by comparing the measured TC concentrations with the theoretical values.

### Comparison with other total cholesterol measurement methods

The CEM was measured using a widely reported method to compare our method with a conventional approach. The erythrocyte membranes were isolated by hypotonic lysis. Briefly, packed erythrocytes were diluted 50-fold with 5 mM Na_2_HPO_4_ (pH 8.0) and vortexed vigorously. The resulting erythrocyte ghosts were collected by centrifugation at 20,630×g for 30 min at 4°C. The pellet was washed thrice with 5 mM Na_2_HPO_4_ (pH 8.0) by centrifugation under the same conditions to remove residual hemoglobin. The final membrane pellet was resuspended in a small volume of phosphate-buffered saline. TC and protein concentrations of the isolated membranes were determined using enzymatic assays and the Lowry method, respectively [16]. CEM was calculated as the ratio of TC to protein content (µg cholesterol/mg protein).

To confirm the accuracy of the direct method, the TC content of erythrocytes was determined using the Abell–Kendall (AK) method [17]. The original AK method was modified by reducing the reaction volume five-fold.

### Measurement of clinical laboratory data

Complete blood cell counts were determined using an automated hematology analyzer XN-9100 (Sysmex, Kobe, Japan). The following parameters were obtained: erythrocyte count, hematocrit, hemoglobin concentration, mean corpuscular hemoglobin concentration (MCHC), mean corpuscular volume (MCV), and reticulocyte ratio. Serum lipid profile, including the parameters TC, HDL-C, LDL-C, and triglyceride (TG), was measured using commercial enzymatic assay kits on a Labospect 008α (Hitachi High-Tech Co., Tokyo, Japan). Liver function tests (alanine aminotransferase, gamma-glutamyl transferase, and lactate dehydrogenase) and renal function tests (creatinine and urea nitrogen) were performed on serum samples from 12 healthy participants using standard laboratory methods. Hemoglobin A1c (HbA1c) levels were quantified by HPLC using an automated glycohemoglobin analyzer HLC-723G11 (Tosoh, Tokyo, Japan).

### Preparation of erythrocytes fraction with different densities

Two milliliters of washed erythrocytes were gently placed into 3 ml of saline in small-diameter tubes, allowing them to settle slowly through the saline solution. The tubes were centrifuged at 2,000×g for 5 min at 4°C without inversion to create a density gradient. The upper saline layer was carefully removed after centrifugation. The erythrocyte layer was then fractionated into 10 aliquots of 200 μl each, starting from the top of the gradient. The erythrocyte TC content per volume and per cell in these fractions was measured using a previously described method.

### Statistics

**Figure 1:**
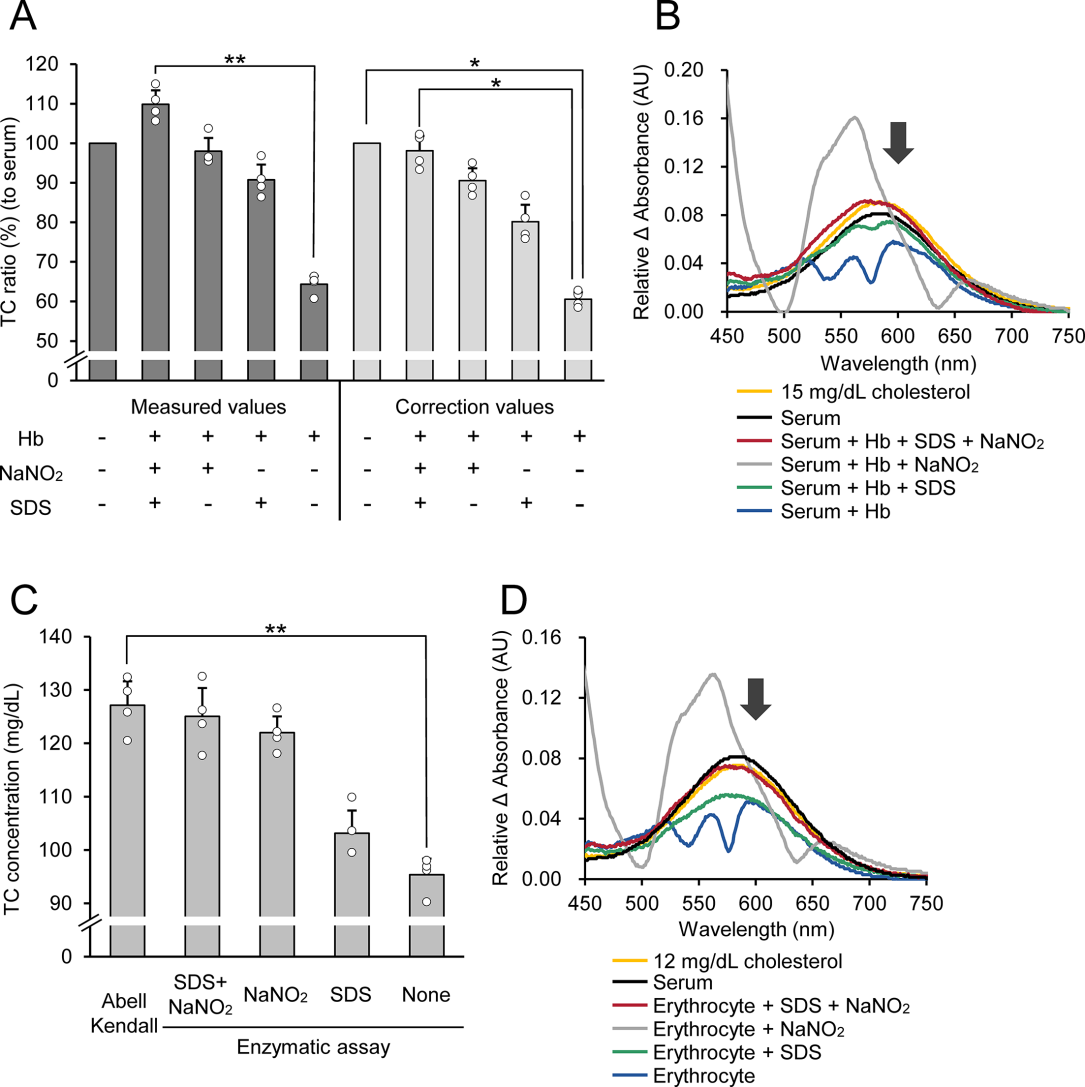
Verification of hemoglobin interference avoidance and measurement of erythrocyte cholesterol concentration. (**A**) Comparison of cholesterol concentrations in diluted serum with and without the addition of 3,500 mg/dl hemoglobin. Cholesterol concentrations were measured in the presence of hemoglobin alone, hemoglobin treated with SDS, hemoglobin treated with NaNO_2_, and hemoglobin treated with both SDS and NaNO_2_. Correction values were calculated by subtracting the cholesterol content of the hemoglobin solution (cholesterol bound to hemoglobin) from the measured cholesterol concentration. Data are represented as mean ± SD (*n* = 4, different individuals), and open circles show individual points. Friedman’s test with Bonferroni correction as a post hoc test was used for multiple comparisons. *P < 0.05, **P < 0.01. (**B**) Representative absorption difference spectra of serum samples with and without hemoglobin addition. The spectra were obtained in the presence of hemoglobin alone, hemoglobin treated with SDS, hemoglobin treated with NaNO_2_, and hemoglobin treated with both SDS and NaNO_2_. Representative data from samples from four individuals are presented. For comparison, the spectra of serum and cholesterol standard solutions are also shown. The arrow indicates the wavelength at which cholesterol concentration was measured. (**C**) Comparison of erythrocyte cholesterol concentrations measured using the developed method with those measured using the Abell-Kendall method. Data are presented as mean ± SD (*n* = 4, different individuals), and open circles show individual points. Friedman’s test with Bonferroni correction as a post hoc test was used for multiple comparisons. **P < 0.01. (**D**) Representative absorption difference spectra of erythrocytes treated with different combinations of denaturants: SDS alone, NaNO_2_ alone, or both SDS and NaNO_2_. Representative data from four individuals are presented. For comparison, the spectra of serum and cholesterol standard solutions are also shown. The arrow indicates the wavelength at which cholesterol concentration was measured. Hb, hemoglobin; NaNO_2_, sodium nitrite; SDS, sodium dodecyl sulfate.

**Figure 2:**
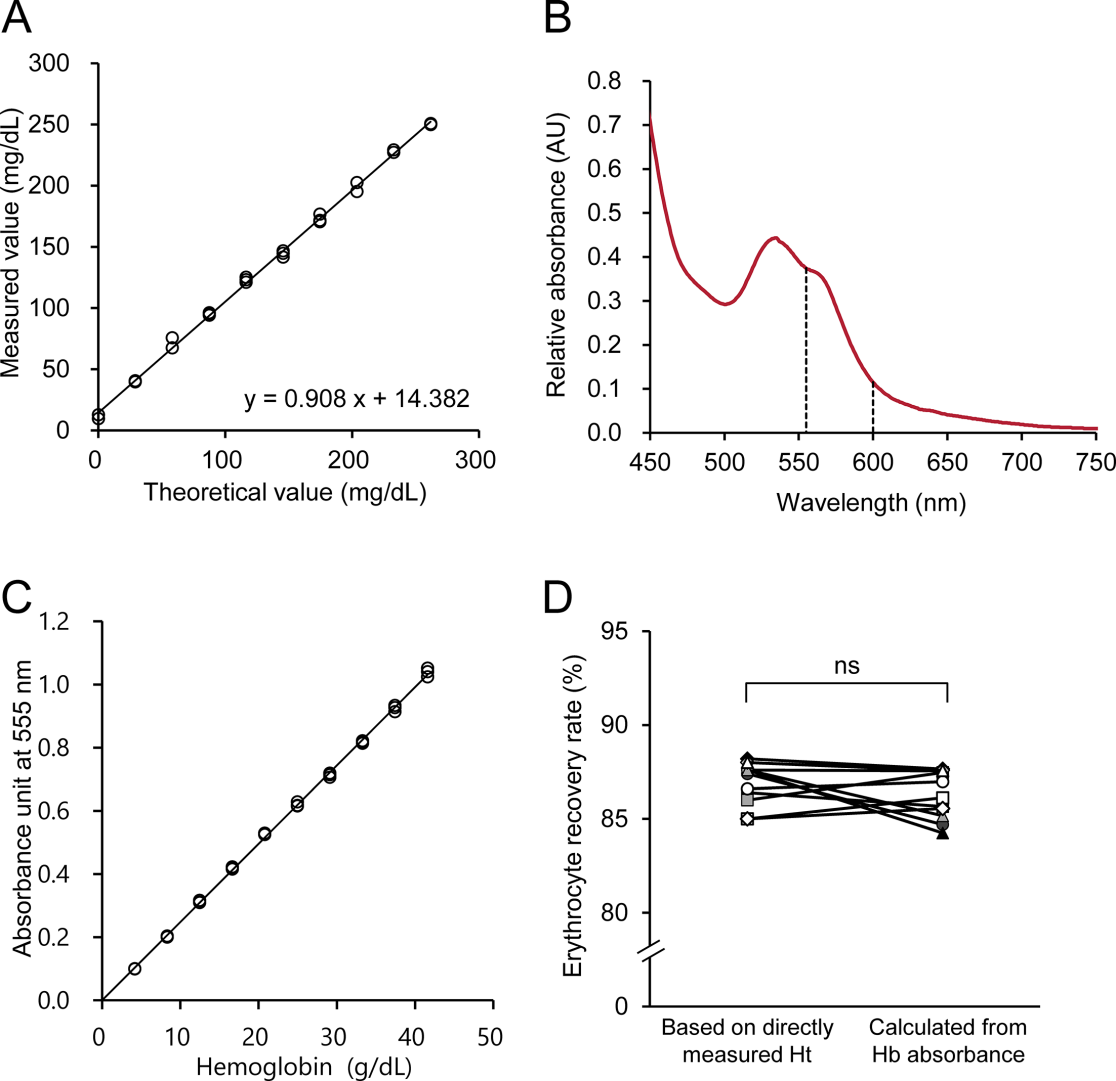
Validation and correction of erythrocyte cholesterol concentration. (**A**) Linearity of the cholesterol measurement assay. Each datapoint represents the cholesterol concentration measured in triplicate samples spiked with various concentrations of cholesterol standard solutions at a final hemoglobin concentration of 3,500 mg/dl. (**B**) Representative absorption spectra of erythrocytes treated with SDS and NaNO_2_. Representative data from four individuals are presented. (**C**) Linearity of hemoglobin absorbance measurements. Hemoglobin absorbance at 555 nm was measured. (**D**) Comparison of the erythrocyte recovery rates. Each datapoint represents the recovery rate of an individual, calculated from either hemoglobin absorbance or the direct measurement of hematocrit. Datapoints from the same individual are connected by lines (*n* = 12). ns, not significant (paired t-test). Hb, hemoglobin; Ht, hematocrit.

**Figure 3:**
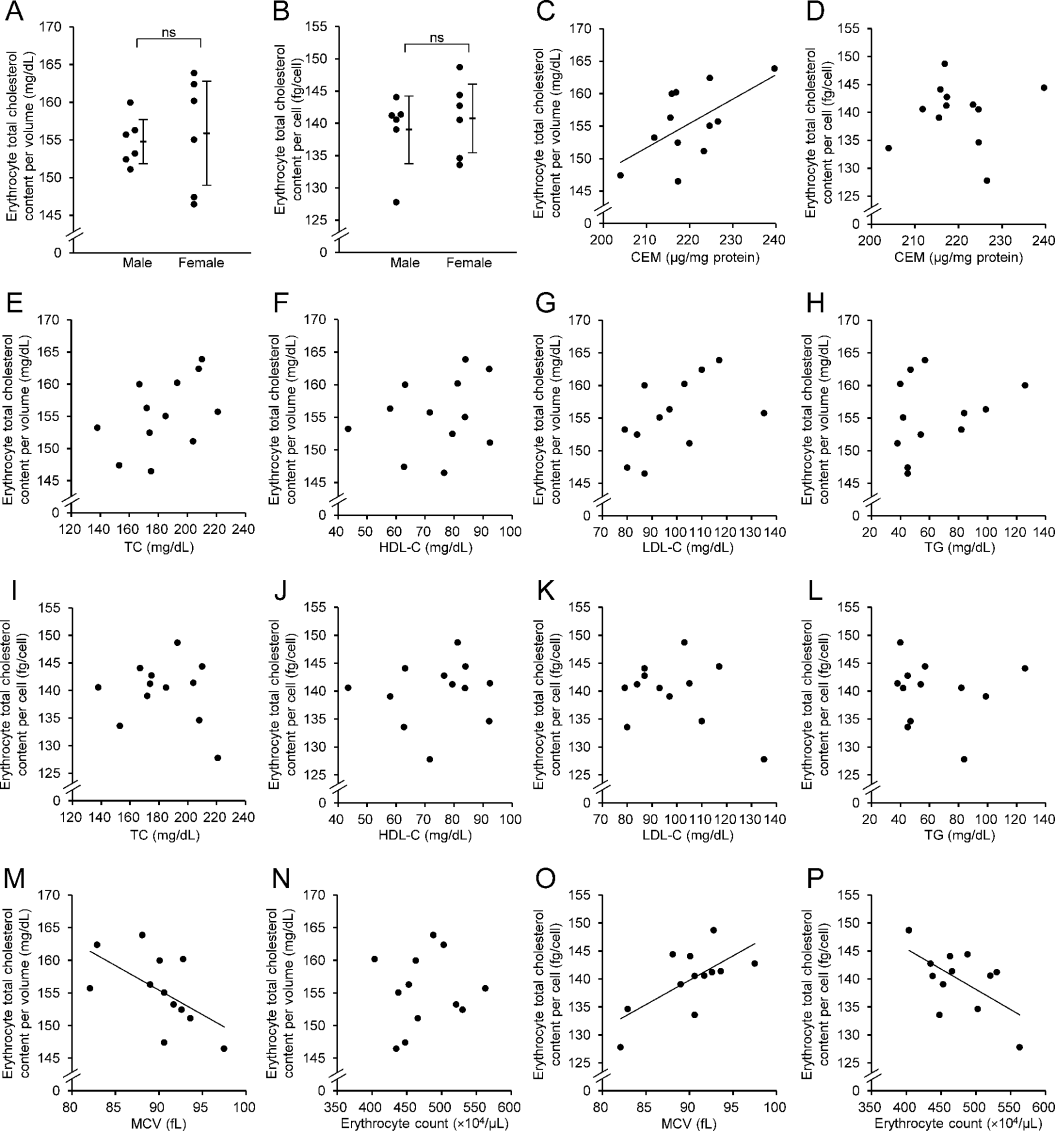
Comparison and correlation of erythrocyte cholesterol content. (**A & B**) Comparison of erythrocyte cholesterol content (per volume [mg/dl] and per cell [fg/cell]) between men and women. The Mann–Whitney *U* test was used to compare two groups. ns, not significant. (**C & D**) Correlation between erythrocyte cholesterol content (per volume and per cell) and CEM. (**E-L**) Correlation between erythrocyte cholesterol content (per volume and per cell) and serum lipid parameters, including TC, HDL-C, LDL-C, and TG. (**M-P**) Correlation between erythrocyte cholesterol content (per volume and per cell) and erythrocyte parameters, including MCV and erythrocyte count. Spearman’s rank correlation test was used only for correlations with TG levels, while Pearson’s correlation test was used for all other correlations. CEM, cholesterol content of the erythrocyte membrane; HDL-C, high-density lipoprotein cholesterol; LDL-C, low-density lipoprotein cholesterol; MCV, mean corpuscular volume; TC, total cholesterol; TG, triglycerides.

**Figure 4:**
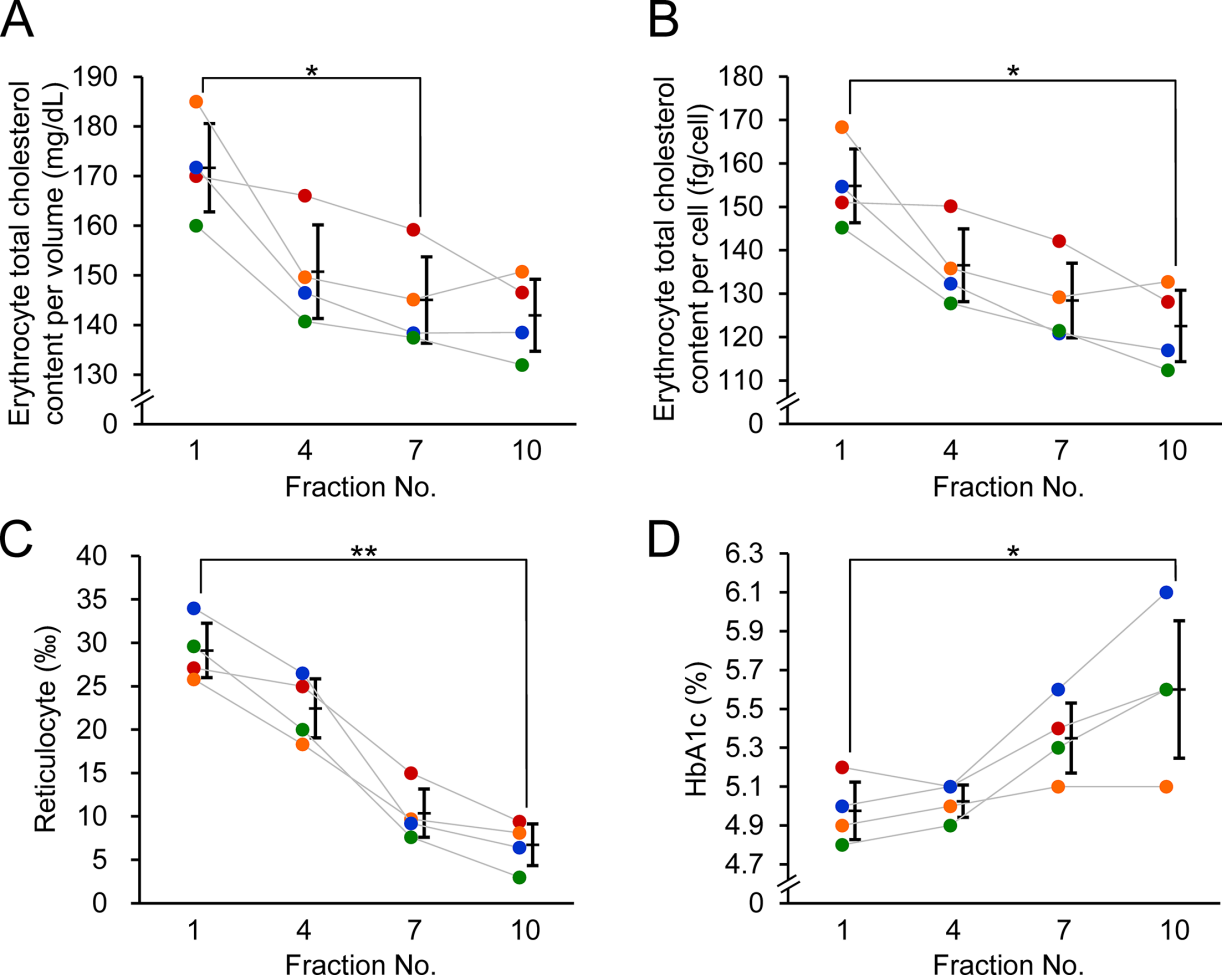
Intra-individual variation of erythrocyte cholesterol content across different density fractions. (**A**) Erythrocyte cholesterol content per volume (mg/dl), (**B**) Erythrocyte cholesterol content per cell (fg/cell), (**C**) Reticulocyte ratio (‰), and (**D**) HbA1c (%) in different density fractions were compared using Friedman’s test with Bonferroni correction for post hoc analysis. *P < 0.05, **P < 0.01.

All data are presented as mean ± SD. All statistical analyses were performed using SPSS version 25.0 (IBM Corp., Armonk, NY, U.S.A.). The Kolmogorov–Smirnov test was used to assess the normality of data distribution. Nonparametric tests were employed for data that did not conform to a Gaussian distribution or for groups with small sample sizes (*n* ≤ 4). Friedman’s tests with Bonferroni correction as a post hoc test for multiple comparisons were used to analyze the differences between different experimental conditions or between erythrocyte fractions, as indicated in(Figures 1 and 4). Comparisons of erythrocyte recovery rates were assessed using paired *t*-tests (Figure 2D). Differences between men and women participants were evaluated using the Mann–Whitney *U* test (Figure 3A and B). Pearson’s correlation test or Spearman’s rank correlation test was used to assess the correlations between variables, as shown in Figure 3C–P. Statistical significance was set at *P*<0.05.

## Results

### Verification of avoidance of the hemoglobin interference

To evaluate the influence of hemoglobin on the TC measurement assay, we assessed the accuracy of TC measurement in serum samples with or without 3,500 mg/dl hemoglobin, a concentration approximating one-tenth of the MCHC. The serum samples were diluted 10-fold, resulting in a TC concentration comparable to that obtained after diluting packed erythrocytes 10-fold. In the presence of hemoglobin, the measured TC concentration in serum decreased to 64.3 ± 2.1% of the value obtained in the absence of hemoglobin (Figure 1A). To mitigate the negative effects of hemoglobin, we investigated the use of SDS and NaNO_2_ as hemoglobin denaturants. When only SDS was added to the hemoglobin-containing serum, the measured TC concentration remained lower, at 90.8 ± 3.8% of the control value. In contrast, the addition of NaNO_2_ alone resulted in a TC concentration of 98.0 ± 3.3%, similar to that observed in the absence of hemoglobin. The combination of SDS and NaNO_2_ further improved the TC value, resulting in a concentration of 109.9 ± 3.5%, exceeding the control value. To correct for any potential interference from cholesterol associated with hemoglobin, the TC content of the hemoglobin solutions was measured under each condition and subtracted from the measured TC values. After correction, TC concentrations in the presence of SDS alone and NaNO_2_ alone were low at 80.2 ± 4.3% and 90.6 ± 3.1% relative to the control, respectively, while the combination of SDS and NaNO_2_ resulted in 98.1 ± 3.8%, which is close to the value in serum. Furthermore, we examined the effect of hemoglobin on the absorption spectra of the reaction mixtures. In the presence of hemoglobin, the absorption difference spectra were significantly distorted (Figure 1B). The distortion was more pronounced when hemoglobin was treated with NaNO_2_ alone. While the addition of SDS alone partially alleviated the spectral distortion, the combination of NaNO_2_ and SDS effectively eliminated the interference from hemoglobin.

### Measurement of erythrocyte total cholesterol concentration

To validate the accuracy of our method, we compared the cholesterol content of erythrocytes measured using our novel method to that obtained using the AK method, which is considered the gold standard for cholesterol determination. In samples treated with both SDS and NaNO_2_, the measured erythrocyte cholesterol concentration (125.0 ± 5.3 mg/dl) was not significantly different from that determined using the AK method (127.1 ± 4.5 mg/dl) (Figure 1C). In contrast, cholesterol concentrations measured in samples treated with NaNO_2_ alone (122.0 ± 3.1 mg/dl), without any denaturant (95.4 ± 3.0 mg/dl), or with SDS alone (103.2 ± 4.2 mg/dl) were lower than those measured using the AK method. Consistent with the findings of the hemoglobin interference experiments, the addition of both SDS and NaNO_2_ effectively eliminated the distortion of the difference spectra, as shown in Figure 1D.

### Validation of the new method for erythrocyte cholesterol concentration

To validate the performance of the new direct measurement method, we evaluated its intra-assay repeatability, between-day precision, linearity, and accuracy using a spike-recovery test. The coefficient of variation (CV) was calculated to assess the intra-assay repeatability and between-day precision (Tables 1 and 2). The CV for intra-assay repeatability ranged from 3.3% to 4.1%, whereas that for between-day precision ranged from 3.2% to 3.8%. At a hemoglobin concentration of 3,500 mg/dl, linearity was observed up to a cholesterol concentration of approximately 250 mg/dl (Figure 2A). Additionally, we performed a spike-recovery test by adding various concentrations of cholesterol standard solutions to the packed erythrocytes and measuring the cholesterol concentrations in the resulting samples (Table 3). When cholesterol was spiked at concentrations ranging from 50 to 100 mg/dl, the recovery rates were between 96.5% and 98.9%.

**Table 1:** Intra-assay repeatability

	Sample 1	Sample 2	Sample 3
Mean (mg/dl)	114.1	118.5	124.0
SD (mg/dl)	4.5	4.8	4.1
CV (%)	3.9	4.1	3.3

SD, standard deviation; CV, coefficient of variation.

**Table 2:** Between-day precision

	Sample 1	Sample 2	Sample 3
Mean (mg/dl)	127.2	128.2	130.0
SD (mg/dl)	4.8	4.1	4.3
CV (%)	3.8	3.2	3.3

SD, standard deviation; CV, coefficient of variation.

**Table 3:** Spike-recovery test

Measured value (mg/dl)	120.4	169.8	192.7	218.2
Added value (mg/dl)	0.0	50.0	75.0	100.0
Recovered value (mg/dl)		49.4	72.3	97.8
Recovery rate (%)		98.9	96.5	97.8

### Correction by simultaneous measurement of hemoglobin concentration

During the preparation of packed erythrocytes, it is challenging to completely remove the residual solvents. To accurately quantify erythrocyte TC content, it is crucial to correct for variations in erythrocyte recovery. Therefore, we developed a method to correct for erythrocyte recovery by simultaneously measuring hemoglobin absorbance at 555 nm, which corresponds to a shoulder peak in the absorption spectrum of hemoglobin, after adding the first reagent in the TC measurement assay (Figure 2B). We confirmed the linearity of hemoglobin absorbance at 555 nm by measuring a series of hemoglobin solutions with known concentrations using Interference Check A Plus (Sysmex Corporation, Japan) (Figure 2C). Erythrocyte recovery was calculated based on the hemoglobin concentration measured simultaneously with the cholesterol concentration and MCHC of whole blood using the following equation:

*Erythrocyte recovery rate* (%) = (*Hb concentration of washed erythrocytes* measured by absorbance at 555 nm/*MCHC of whole blood*) × 100.

To assess the validity of this method, we compared the erythrocyte recovery calculated based on hemoglobin absorbance with that determined directly by measuring the hematocrit of the packed erythrocytes diluted two-fold with saline. No significant differences were observed between the two methods (Figure 2D). These findings demonstrate that the simultaneous measurement of hemoglobin absorbance provides an accurate method for correcting erythrocyte recovery and improving the accuracy of erythrocyte cholesterol quantification.

### Measurement in healthy subjects and calculation of cholesterol amount per volume and per cell

To establish a reference range, we measured erythrocyte TC in 12 healthy volunteers (six men and six women) using the newly developed method. Participants were considered healthy if their lipid concentrations (TC, HDL-C, LDL-C, and TG) and liver and kidney function test results were within the normal ranges (Table 4). Erythrocyte TC concentration was expressed as TC content per volume (mg/dl) and calculated by dividing the measured cholesterol value by the erythrocyte recovery rate. Subsequently, the erythrocyte TC content per cell (fg/cell) was calculated by correcting the TC concentration using whole blood hematocrit and erythrocyte counts using the following equations:

1. *Erythrocyte TC content per volume* (mg/dl) = *Measured erythrocyte TC value* (mg/dl) / *Erythrocyte recovery rate*
2. *Erythrocyte TC content per cell* (fg/cell) = *Erythrocyte TC content per volume* (mg/dl) / (*Whole blood erythrocyte count* ( × 10^13^ /L) /*Whole blood hematocrit* (%) × 100).

**Table 4:** 

Profile	
Erythrocyte total cholesterol content per volume (mg/dl)	155.3 ± 5.3
Erythrocyte total cholesterol content per cell (fg/cell)	139.9 ± 5.3
CEM (µg/mg protein)	219.8 ± 8.5
TC (mg/dl)	183.3 ± 23.8
HDL-C (mg/dl)	74.1 ± 14.1
LDL-C (mg/dl)	98.1 ± 16.1
TG (mg/dl)	63.3 ± 26.9
Erythrocyte count ( × 10^4^ /µl)	476 ± 44
Hb (g/dl)	14.1 ± 1.3
Ht (%)	42.8 ± 3.3
MCV (fL)	90.1 ± 4.1
MCH (pg)	29.7 ± 1.6
MCHC (mg/dl)	33.0 ± 0.8
Reticulocyte (‰)	14.5 ± 3.7

CEM, cholesterol content of erythrocyte membrane; TC, total cholesterol; HDL-C, high-density lipoprotein cholesterol; LDL-C, low-density lipoprotein cholesterol; TG, triglyceride; Hb, hemoglobin; Ht, hematocrit; MCV, mean corpuscular volume; MCH, mean corpuscular hemoglobin; MCHC, mean corpuscular hemoglobin concentration.

The mean erythrocyte TC content per volume was 154.8 ± 2.9 mg/dl in men and 155.9 ± 6.9 mg/dl in women (Figure 3A). The mean erythrocyte TC content per cell was 139.0 ± 5.2 fg/cell in men and 140.8 ± 5.3 fg/cell in women (Figure 3B). No significant differences in erythrocyte TC contents per volume or cell were observed between men and women.

### Comparison with conventional method, serum lipid tests, and erythrocyte-related parameters

To investigate the relationship between erythrocyte cholesterol content measured using our method and other relevant parameters, we compared our results with CEM, serum lipid profiles, and erythrocyte-related parameters in samples from healthy subjects. The erythrocyte TC content per volume was significantly positively correlated with CEM (*r* = 0.598, *P*=0.040) (Figure 3C). In contrast, no significant correlation was observed between the erythrocyte TC content per cell and CEM (*r* = 0.066, *P*=0.840) (Figure 3D). No significant correlations were found between the erythrocyte TC concentration (per volume or per cell) and serum lipid parameters, including TC, HDL-C, LDL-C, and TG (Figures 3E–L). The erythrocyte TC content per volume showed a significant negative correlation with MCV (*r* = -0.583, *P*=0.047), whereas no correlation was observed with the erythrocyte count ((Figures 3M and 3N). In contrast, the erythrocyte TC content per cell was positively correlated with MCV (*r* = 0.673, *P*=0.016) and negatively correlated with erythrocyte count (*r* = −0.597, *P*=0.040) (Figures 3O and 3P).

### Intra-individual variation of erythrocyte cholesterol contents in different density fractions

To investigate the TC content in erythrocytes at different stages of maturation, we prepared erythrocyte fractions based on density. Erythrocyte TC content was measured in the first, fourth, seventh, and tenth fractions, with the first fraction representing the least dense (youngest) erythrocytes and the tenth fraction representing the densest (oldest) erythrocytes. Both erythrocyte TC content per volume and per cell decreased with increasing erythrocyte density. The erythrocyte TC content per volume in the seventh fraction was significantly lower than that in the first fraction (*P*=0.037) (Figure 4A), and the erythrocyte TC content per cell in the tenth fraction was significantly lower than that in the first (*P*=0.016) (Figure 4B). These results demonstrate that our newly developed method can reflect differences in erythrocyte TC content based on cell age. This was supported by the observation that the reticulocyte ratio, an indicator of young erythrocyte abundance, was significantly higher in the first fraction than in the tenth fraction (*P*=0.006) (Figure 4C). This was also observed for HbA1c, which increased with erythrocyte age, showing a significant increase across the density gradient, with the highest levels observed in the densest (tenth) fraction (*P*=0.024) (Figure 4D).

## Discussion

Erythrocyte cholesterol content has emerged as a potentially important biomarker of ASCVD. However, its clinical utility is limited by the complexity of conventional measurement techniques. This study aimed to develop a simple and clinically applicable method for measuring erythrocyte cholesterol content, thereby enabling the assessment of residual ASCVD risk, which cannot be fully captured by HDL-C or LDL-C measurements alone.

To achieve this, we investigated the feasibility of directly mixing erythrocytes containing hemoglobin with a cholesterol reagent for the enzymatic assay. Preliminary experiments demonstrated that high concentrations of hemoglobin interfered with the absorbance caused by quinone pigments produced during enzymatic reactions, resulting in an underestimation of cholesterol levels. To address this issue, we added SDS and NaNO_2_, which facilitate hemoglobin denaturation and methemoglobin formation, respectively. NaNO_2_ is known as an oxidizing agent that generates methemoglobin [18], and was initially added to shift the absorption wavelength of hemoglobin. However, its effect was insufficient in terms of the shape of the difference spectrum. SDS, also known as sodium lauryl sulfate in hemoglobin measurement [19], added in addition to NaNO_2_, has the effect of solubilizing cell membranes. These denaturants effectively eliminated the negative effects of hemoglobin on cholesterol measurements. However, the observed cholesterol concentrations were unexpectedly elevated, likely due to cholesterol binding to hemoglobin, as reported in previous studies [6,20].

A notable limitation of existing erythrocyte cholesterol measurement methods is the lack of validation for accuracy and basic performance metrics [10,13,14]. In this study, we addressed this gap by conducting a comprehensive validation. Cholesterol concentrations measured using the newly developed method were consistent with those obtained using the AK method and closely aligned with previously reported values derived from extraction-based approaches [6,7]. Furthermore, the method demonstrated good performance in terms of intra-assay repeatability, between-day precision, linearity, and spike-recovery tests.

We measured the erythrocyte cholesterol content in packed erythrocytes from 12 healthy participants to determine the reference range and compare the results with conventional lipid biomarkers. A correlation with the CEM was observed, indicating that the values obtained using our method were comparable with those in previous reports [10–15]. In contrast, no correlation was found with serum lipid levels, suggesting that the erythrocyte cholesterol content may represent a distinct biomarker with unique clinical implications. Additionally, as erythrocyte cholesterol levels increased depending on cell size, the observed values were considered reasonable.

Erythrocyte density increases with cell aging [21,22]. Leveraging this property, we prepared density-gradient erythrocyte fractions to assess the erythrocyte cholesterol content at various maturation stages. As expected, reticulocytes, which are relatively immature erythrocytes released from the bone marrow into the peripheral blood for a few days, were enriched in the upper fractions, whereas their number decreased toward the bottom fraction, suggesting that these fractions contained erythrocytes at different maturation stages. HbA1c levels, which increase with erythrocyte aging, further validated this separation [23]. Our results revealed that the cholesterol content, both per volume and per cell, tended to decrease in older erythrocytes. This finding is consistent with previous studies utilizing high-resolution mass spectrometry, which demonstrated higher absolute cholesterol content in reticulocytes than in mature erythrocytes [24], supporting the validity of our method.

Regarding the relationship between erythrocyte age and ASCVD, it is well-established that aging erythrocytes exhibit reduced deformability [25,26], which may contribute to an increased risk of ASCVD. In contrast, a recent study reported an inverse association between reticulocyte levels and atherosclerosis [27]. These findings collectively suggest that lower erythrocyte cholesterol content is likely associated with the progression of ASCVD. Although further clinical studies are needed to elucidate the precise relationship between erythrocyte cholesterol content and ASCVD, our findings suggest that, unlike previous studies, evaluating erythrocyte cholesterol content may be more informative when considering erythrocyte maturation stages rather than relying solely on total erythrocyte cholesterol levels.

One limitation of this study is that it exclusively involved healthy individuals, leaving the extent of variation in erythrocyte cholesterol content in patient samples unclear. Further investigations using patient samples are necessary to assess the clinical relevance of erythrocyte cholesterol levels in predicting ASCVD risk. Additionally, intra-individual variability in erythrocyte cholesterol content influenced by lipoprotein-mediated cholesterol exchange should be explored in future studies involving patients with altered lipoprotein profiles [8]. Another limitation is that erythrocyte cholesterol was measured as total amounts without distinguishing between free and esterified forms. However, in erythrocytes, most of the cholesterol is free, with minimal esterified cholesterol [28], and there is neither synthesis nor esterification of cholesterol [29]. Therefore, the measured TC in erythrocytes can largely be considered as a quantity reflecting free cholesterol. Regarding this point, further studies are needed on direct measurement methods for distinguishing between free and esterified cholesterol and on the clinical significance of measuring them separately.

In conclusion, we successfully established a novel method for quantifying erythrocyte cholesterol content that overcomes the interference caused by hemoglobin using SDS and NaNO_2_. This method enables the straightforward quantification of erythrocyte cholesterol content per volume and per cell, offering potential as a new biomarker for clinical applications.

## Supplementary material

Online supplementary figure 1

Online supplementary figure 2

## Data Availability

The data used to support the findings of the present study are available from the corresponding author (Ryunosuke Ohkawa, Graduate School of Medical and Dental Sciences, Institute of Science Tokyo, ohkawa.alc@tmd.ac.jp) upon request.

## Open Access

This article has been published open access under our Subscribe to Open programme, made possible through the support of our subscribing institutions, learn more here: https://portlandpress.com/pages/open_access_options_and_prices#conditional

## Abbreviations

CEM: cholesterol content of erythrocyte membrane
HDL-C: high-density lipoprotein cholesterol
Hb: hemoglobin
Ht: hematocrit
LDL-C: low-density lipoprotein cholesterol
MCV: mean corpuscular volume
TC: total cholesterol
TG: triglyceride
